# Splicing- and demethylase-independent functions of LSD1 in zebrafish primitive hematopoiesis

**DOI:** 10.1038/s41598-020-65428-9

**Published:** 2020-05-22

**Authors:** Junya Tamaoki, Miki Takeuchi, Ryo Abe, Hiroshi Kaneko, Taeko Wada, Shinjiro Hino, Mitsuyoshi Nakao, Yusuke Furukawa, Makoto Kobayashi

**Affiliations:** 1 Department of Molecular and Developmental Biology, Faculty of Medicine, University of Tsukuba, Tsukuba, 305-8575 Japan; 2 Division of Stem Cell Regulation, Center for Molecular Medicine, Jichi Medical University, Shimotsuke, 329-0498 Japan; 3 Department of Medical Cell Biology, Institute of Molecular Embryology and Genetics, Kumamoto University, Kumamoto, 860-0811 Japan

**Keywords:** Histone post-translational modifications, Haematopoiesis, RNA splicing, Zebrafish

## Abstract

LSD1/KDM1A is a widely conserved lysine-specific demethylase that removes methyl groups from methylated proteins, mainly histone H3. We previously isolated the zebrafish LSD1 gene and demonstrated that it is required for primitive hematopoiesis. Recently, a neuron-specific splicing variant of LSD1 was found in mammals and its specific functions and substrate specificities were reported. To our surprise, zebrafish LSD1 cDNA, which we previously analyzed, was corresponded to the neuron-specific variant in mammals. In this study, we investigated the structures and expression of LSD1 splicing variants in zebrafish and found all 4 types of LSD1 isoforms: LSD1, LSD1+2al, LSD1+8al and LSD1+2al8al. Interestingly, LSD1+8al/LSD1+2al8al, which correspond to mammalian neuron-specific variants, expressed ubiquitously in zebrafish. We also performed phenotypic rescue experiments of a zebrafish LSD1 mutant (*kdm1a*^*it627*^) using human and zebrafish LSD1 variants to identify which variant is involved in primitive hematopoiesis. Unexpectedly, the overexpression of all types of human and zebrafish variants was able to rescue the hematopoietic phenotypes in LSD1 mutants. Furthermore, enzymatic-deficient LSD1K661A (human) and K638A (zebrafish) were also able to rescue the mutant phenotypes. These results suggest that the LSD1 functions in zebrafish primitive hematopoiesis are free from any splicing-dependent regulation or demethylation reaction.

## Introduction

The first histone demethylase LSD1 (also known as KDM1A) was discovered in 2004 and shown to catalyze the selective oxidative demethylation of histone H3 in a flavin adenine dinucleotide-dependent manner^1^. Phylogenetic analyses have been shown that LSD1 is widely conserved among eukaryotes, except for some fungi, such as the budding yeast *Saccharomyces cerevisiae*^2^. LSD1 participates in the widespread epigenetic regulations of both normal and disease state transcriptional programs, such as hematopoietic and neuronal differentiation, animal development, and cancer proliferation and metastasis^3,4^. The primary substrate of LSD1 was initially identified as mono- or di-methylated lysine 4 of histone H3 (H3K4), while other physiological substrates have also been reported, such as methylated-lysine 9 of histone H3^5^, p53^6^, and Dnmt1^7^. Interestingly, a neuron-specific splicing variant of LSD1, named LSD1+8a or neuroLSD1, which contains micro-exon 8a, was identified in humans and mice^8^ and has recently been shown to have a different substrate preference from the ubiquitously expressed LSD1 isoform, which has no extra exons^9,10^. Furthermore, another splicing variant known as LSD1+2a, which contains micro-exon 2a, was also found in humans and mice^8^, demonstrating an enzymatic activity that was significantly weaker than that of the isoform without micro-exons^11^. Since the expression of LSD1+8a is developmentally regulated and neural activity-dependent^12^, while that of LSD1+2a is differentiation-dependent in hematopoietic stem/progenitor cells^11^, a variety of LSD1 functions may be generated and regulated by alternative splicing.

**Figure 1:**
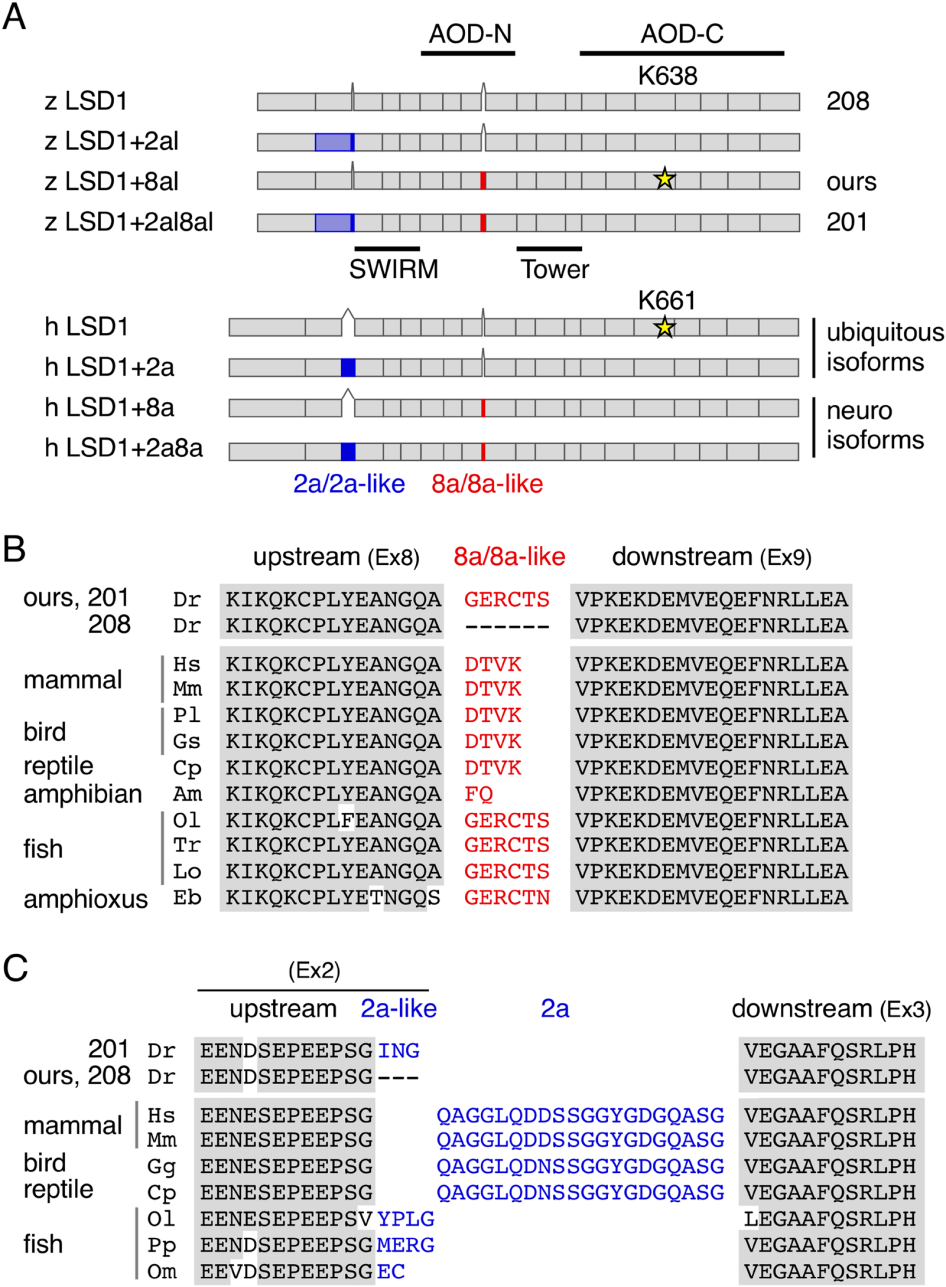
Splicing variants similar to LSD1+2a and LSD1+8a in vertebrates. (**A**) Schematic images of zebrafish (z) and human (h) LSD1 splicing variants. AOD-N, AOD-C, SWIRM, and Tower denote the N-terminal and C-terminal AOD; the Swi3p, Rsc8p and Moira domain; and the helical tower domain, respectively. K638 and K661 with yellow stars are crucial catalytic residues in zebrafish and human LSD1, respectively. (**B**) A comparison of amino acid sequences of LSD1 8a/8a-like peptides and their up- and down-stream sequences (corresponding to exons 8 and 9, respectively, in human LSD1) among vertebrates. Abbreviations and accession numbers: Am, *Ambystoma mexicanum* (axolotl), JK979304; Cp, *Chrysemys picta* (turtle), XM_005309376; Gs, *Gavia stellata* (loon), XM_009816565; Dr, *Danio rerio* (zebrafish), EB917944 and EB927844; Eb, *Eptatretus burger* (hagfish), ENSEBUT00000024774.1; Hs, *Homo sapiens* (human), AA127204; Lo, *Lepisosteus oculatus* (gar), XM_006631419; Mm, *Mus musculus* (mouse), BQ443150; Ol, *Oryzias latipes* (medaka), XM_011490201; Pl, *Phaethon lepturus* (tropicbird), XM_010287337; Tr, *Takifugu rubripes* (fugu**)**, XM_011618380. (**C**) A comparison of amino acid sequences of LSD1 2a/2a-like peptides and their up- and down-stream sequences (corresponding to exons 2 and 3, respectively, in human LSD1) among vertebrates. Abbreviations and accession numbers: Cp, XM_005309377; Dr, EB917944 and AI629331;Gg, *Gallus gallus* (chicken), XM_417719; Hs, NM_001009999; Mm, NM_001347221; Om, *Oncorhynchus mykiss* (trout), BX909710; Pp, *Pimephales promelas* (minnow), DT111570.

We previously isolated a zebrafish orthologue gene of mammalian LSD1 (*lsd1/kdm1a*) and demonstrated that it plays critical roles in primitive hematopoiesis using the LSD1 mutant line (*kdm1a*^*it627*^)^13^. We showed that hematopoietic defects in LSD1 mutant embryos was rescued by overexpressing zebrafish LSD1 cDNA. The cDNA used in those rescue experiments was not LSD1 isoform without extra peptides, but rather a short peptide Gly-Glu-Arg-Cys-Thr-Ser (GERCTS) that was inserted within a highly conserved N-terminal amino oxidase domain (AOD-N), as shown in Fig. 1A (z LSD1+8al, ours). To our surprise, the insertion site of the GERCTS peptide in our zebrafish LSD1 matched exactly to LSD1+8a in humans and mice in spite of a differing peptide sequence (DTVK) (Fig. 1B). This prompted us to hypothesize that 1) the LSD1+8a type splicing variant in mammals is also conserved in fish, and 2) this isoform specifically plays roles in primitive hematopoiesis. To test our hypothesis, we analyzed the alternative splicing profiles of zebrafish LSD1 in various conditions and carried out rescue experiments of hematopoietic defects in LSD1 mutant embryos using human LSD1 splicing variants in the present study.

## Results

### Zebrafish LSD1 shares splicing variants with mammals

To investigate splicing variants in zebrafish LSD1, we first searched for them in the zebrafish genomic database at Ensembl Genome Server (http://www.ensembl.org/Danio_rerio) and found two full-length zebrafish LSD1 variants (kdm1a-208 and kdm1a-201), both of which were different from the one we had cloned previously (Fig. 1A, ours). kdm1a-208 is a LSD1 isoform without extra peptides, while kdm1a-201 contains two extra peptides, GERCTS and ING, at the equivalent sites of the mammalian splicing variants LSD1+8a and LSD1+2a, respectively (Fig. 1B,C). Interestingly, the lengths and amino acid sequences of these extra peptides were different from those of the mammalian variants (DTVK and QAGGLQDDSSGGYGDGQASG, respectively)^8^; we therefore called the extra peptides in zebrafish “8a-like peptides” and “2a-like peptides”, respectively. Similarly to mammalian peptide 8a, the 8a-like peptide GERCTS in zebrafish was translated from different exon than those of the up- and down-stream protein sequences (Fig. 1B) while, in contrast to mammalian LSD1+2a, the 2a-like peptide ING in zebrafish was the product of the same exon as the upstream protein sequence (Fig. 1C).

To confirm the presence of these alternative splicing variants in zebrafish, we next searched for them in expressed sequence tag (EST) databases (GenBank, EMBL and DDBJ) using the basic local alignment search tool (BLAST). As a result, cDNA clones of zebrafish LSD1 with and without the 8a-like peptide (GenBank accession numbers: EB917944 and EB927844, respectively) and the 2a-like peptide (CD600395 and AI629331) were all found, suggesting that 8a/8a-like- and 2a/2a-like-type splicing variants are indeed present in zebrafish, as in mammals. We next analyzed the EST databases of other species and found that the alternative splicing in these two sites also existed in birds, reptiles, amphibians, fish, and amphioxus (Fig. 1B,C). These findings suggest that both LSD1 splicing variants with and without 8a/8a- and 2a/2a-like peptides are widely conserved among vertebrates.

### Expression profiles of LSD1 splicing variants in zebrafish

**Figure 2:**
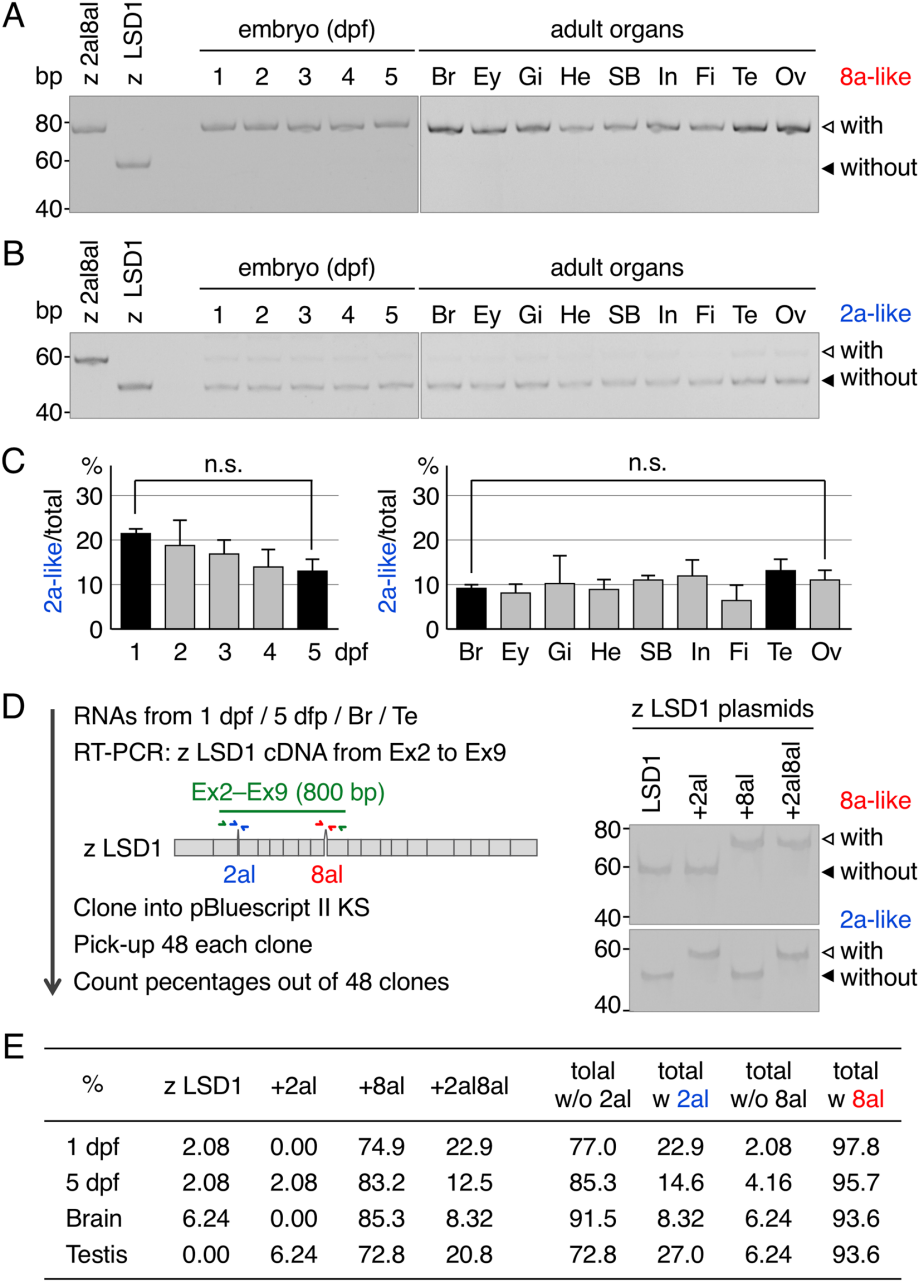
Gene expression profiles of zebrafish LSD1 variants containing 8a- and 2a-like peptides. (**A**) RT-PCR using specific primers (red arrows in panel D) to detect zebrafish LSD1 variants containing 8a-like peptide. The sizes of PCR products with and without 8a-like peptide were 75 bp (open arrowheads) and 57 bp (closed arrowheads), respectively. RNA was extracted from whole embryos at the indicated developmental stages or organs of a 4-month-old adult male, expect for the ovaries (from a 10-month-old adult female). Br, brain; Ey, eye; Gi, gill; He, heart; SB, swim bladder; In, intestine; Fi, fin; Te, testis; Ov, ovary. For positive controls in RT-PCR, pCS2FLlsd1 and pCS2FLlsd1+2al8al were used as templates. (**B**) RT-PCR using specific primers (blue arrows in panel D) to detect zebrafish LSD1 variants containing 2a-like peptide. The sizes of PCR products with and without 2a-like peptide were 56 bp (open arrowheads) and 47 bp (closed arrowheads), respectively. (**C**) Bar graphs indicate the percentages of splicing variants containing 2a-like peptide among total LSD1. Error bars represent standard deviations. The experiments were repeated 3 times. n.s. denotes p values > 0.05. (**D**) Experimental scheme of LSD1 cDNA cloning. Partial LSD1 cDNAs corresponding to exons 2–9 were amplified by RT-PCR using specific primers (green arrows) and indicated total RNAs and cloned into pBluescript II KS. Transformed Forty-eight clones were randomly picked up and analyzed by PCR using specific primers for 2a-like (blue arrows) and 8a-like peptides (red arrows). Right panel indicates the result of control experiments using indicated plasmids. (**E**) Percentages of indicated splicing variants out of 48 clones determined by PCR in panel D.

To examine the expression profile of zebrafish LSD1 variants, we performed reverse transcription-polymerase chain reaction (RT-PCR) using total zebrafish RNA extracted from whole-body embryos at different developmental stages or various adult organs (Fig. 2A–C). The results showed that splicing variants containing 8a-like peptide were expressed in embryos at all developmental stages and in all tested organs of adult fish. This was an unexpected observation since human LSD1 variants with 8a peptide have been shown to be brain- and testis-specific^8^. More surprisingly, we were unable to detect a zebrafish LSD1 variant that did not contain 8a-like peptide under any conditions (closed arrowhead in Fig. 2A), suggesting that it is a minor variant in zebrafish. On the other hand, splicing variants containing 2a-like peptide were expressed at relatively high levels in one day-post fertilization (dpf) embryos, with their levels gradually decreasing during embryonic development, leading to similar levels in all kinds of adult organs (Fig. 1B,C). This expression profile of 2a-like type variants in adult zebrafish was similar with that of human LSD1+2a variant^8^. To confirm these expression profiles of zebrafish LSD1 variants, we isolated and cloned zebrafish LSD1 cDNAs, which contain both of the 2a- and 8a-like splicing sites, from total RNAs prepared from 4 different sources (1-dpf embryos, 5-dpf embryos, adult brain and adult testis)(Fig. 2D). Forty-eight cDNA clones each of 4 different sources were randomly picked up and analyzed the presence of 2a- and/or 8a-like sequences using PCR with 2a- and 8a-like specific primers. As shown in Fig. 2E (total w 2al), percentages of cDNA clones with 2a-like sequences were quite similar with those obtained by RT-PCR (see Fig. 2C). Furthermore, we could isolate cDNAs without 8a-like sequences in all 4 different sources, although their percentages were quite low (2–6%) (Fig. 2E, total w/o 8al), suggesting again that they were indeed minor variants. Importantly, we isolated all 4 types of cDNAs, which encoding LSD1, LSD1+2al, LSD1+8al and LSD1+2al8al, suggesting that all 4 isoforms might exist in zebrafish. Taken together, these results revealed that LSD1 splicing variants containing 8a- and/or 2a-like peptides were both expressed in zebrafish, while the expression profile of zebrafish variants containing 8a-like peptides differs from that of mouse variants containing 8a peptides.

### Impaired primitive hematopoiesis in LSD1 mutant embryos was rescued by the overexpression of any of four splicing variants

**Figure 3:**
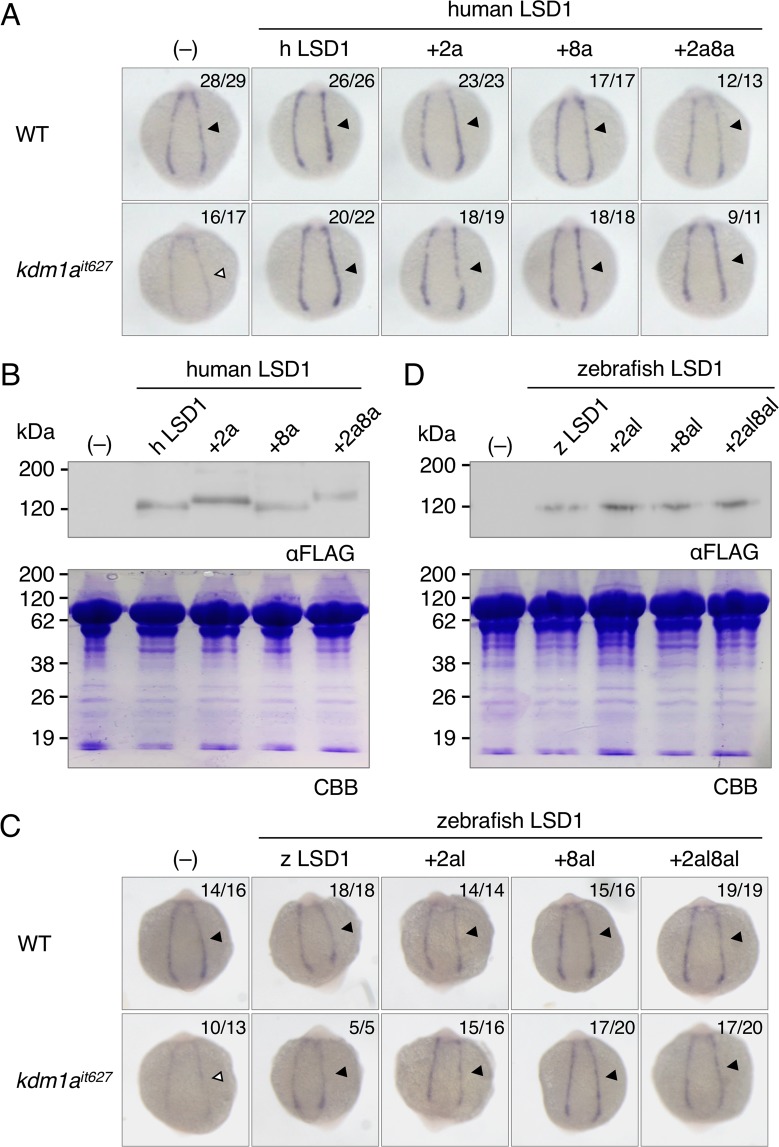
Phenotypic rescue experiments using human and zebrafish LSD1 variants. (**A**) The *gata1a* expression at 15 hpf in the lateral plate mesoderm (arrowheads) of wild-type or *kdm1a*^*it627*^ embryos injected with or without mRNAs encoding indicated human LSD1 variants was detected by WISH analysis. +2a, LSD1+2a; +8a, LSD1+8a; +2a8a, LSD1+2a8a. The numbers in each picture indicate the numbers of embryos with similar staining profiles and tested embryos. WISH was performed in at least two separated trials. (**B**) Immunoblot analysis using anti-FLAG antibody of FLAG-tagged LSD1 variants in 15-hpf wild-type embryos injected with or without mRNAs encoding indicated human LSD1 variants. Total protein loading was shown by CBB staining. (**C**) The *gata1a* expression at 15 hpf in the lateral plate mesoderm (arrowheads) of wild-type or *kdm1a*^*it627*^ embryos injected with or without mRNAs encoding indicated zebrafish LSD1 variants. +2al, LSD1+2al; +8al, LSD1+8al; +2a8al, LSD1+2a8a. (**D**) Immunoblot analysis using anti-FLAG antibody of FLAG-tagged LSD1 variants in 15-hpf wild-type embryos.

Human and mouse LSD1+8a proteins were shown to exhibit a different substrate preference from the LSD1 isoform without 8a peptide, which demethylates di- or mono-methylated histone H3K4^9,10^. Since the zebrafish LSD1 previously used in our rescue experiments was LSD1+8a-like protein^13^, we hypothesized that the functions of LSD1 in zebrafish primitive hematopoiesis required the LSD1+8a-type substrate specificities. To examine this possibility, we performed phenotypic rescue experiments of the downregulated expression of the erythroid marker *gata1a* in LSD1 mutant embryos by overexpressing four human splicing variants: LSD1, LSD1+2a, LSD1+8a, and LSD1+2a8a. The *gata1a* expression in the lateral plate mesoderm at 15 h post fertilization (hpf) was analyzed by whole-mount *in situ* hybridization (WISH) (Fig. 3A), and the protein expression of overexpressed human variants was confirmed by an immunoblot analysis (Fig. 3B). The results showed that the overexpression of any of the four human LSD1 variants was able to rescue the downregulated *gata1a* expression in LSD1 mutant embryos, suggesting that the functions of LSD1 in zebrafish primitive hematopoiesis were splicing-independent. Since sequences of zebrafish 2a- and 8a-like peptides are different from those of mammalian 2a and 8a, it is possible that effects of inserting these peptides on LSD1 functions may differ between zebrafish and mammals. To examine this possibility, we performed phenotypic rescue experiments using zebrafish LSD1 variants: LSD1, LSD1+2al, LSD1+8al, and LSD1+2al8al (Fig. 3C,D). As in the case of human LSD1 variants, overexpression of all four zebrafish LSD1 variants could rescue the downregulated expression of *gata1a* in LSD1 mutant embryos. All these results indicate that LSD1 functions in zebrafish primitive hematopoiesis were splicing-independent.

### Demethylase-inactive LSD1 has a rescue effect on the hematopoietic defects in zebrafish LSD1 mutants

**Figure 4:**
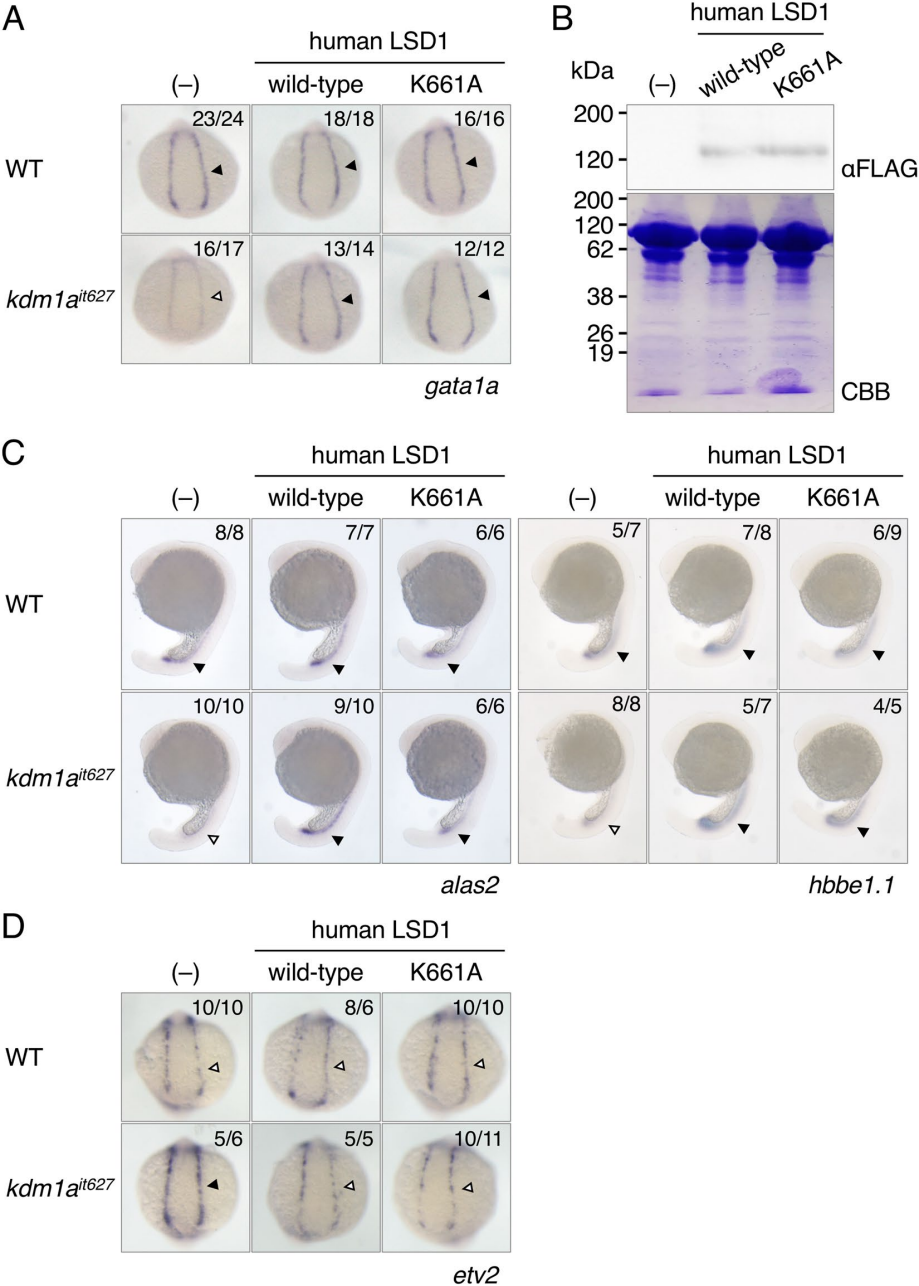
Phenotypic rescue experiments using demethylase-deficient human LSD1 mutant. (**A**) The *gata1a* expression at 15 hpf in the lateral plate mesoderm (arrowheads) of wild-type or *kdm1a*^*it627*^ embryos injected with or without mRNAs encoding indicated wild-type or K661A mutant-type human LSD1 was detected by WISH analysis. (**B**) Immunoblot analysis using anti-FLAG antibody of FLAG-tagged LSD1 proteins in 15-hpf wild-type embryos. Total protein loading was shown by CBB staining. (**C**) Expression of *alas2* and *hbbe1.1*at 20 hpf in the intermediate cell mass (arrowheads). (**D**) Expression of *etv2* at 15 hpf in the lateral plate mesoderm (arrowheads).

We previously showed that the splicing variant LSD1+2a had a lower demethylase activity *in vitro* than LSD1 without micro-exons^11^. Other groups have also reported that human and mouse LSD1+8a and LSD1+2a8a had different substrate preferences from LSD1 isoforms without 8a peptide (histone H3K4-type), such as histones H3K9^9^ or H4K20^10^. One plausible reason for why all four LSD1 variants were able to rescue the hematopoietic defects in LSD1 mutant embryos even though their enzymatic activities and substrate specificities were different is that the functions of LSD1 in zebrafish primitive hematopoiesis may not require its demethylation activity. To test this hypothesis, we used the enzymatic-deficient human LSD1 mutant protein, LSD1K661A in our rescue experiments, since previous reports have shown that introducing an alanine substitution at Lys661 in human LSD1 (K661A) can disrupt its demethylation activity (see Fig. 1A)^14,15^. As we hypothesized, the reduced *gata1a* expression in LSD1 mutant embryos was rescued by the overexpression of human LSD1 K661A to levels similar to those found in wild-type LSD1 (Fig. 4A). An immunoblot analysis showed that the amount of overexpressed LSD1 proteins was equivalent between wild-type LSD1 and K661A mutant (Fig. 4B). Recovery of primitive hematopoiesis in LSD1K661A injected embryos was further confirmed by the expression of erythroid markers, *alas2* and *hbbe1.1* (Fig. 4C). Since we previously demonstrated that the fundamental cause of hematopoietic defects in *lsd1* mutant embryos was the upregulation of *etv2*^13^, we next examined the expression of *etv2* in LSD1 overexpressed embryos (Fig. 4D). The upregulated *etv2* expression in LSD1 mutant embryos was rescued by the overexpression of either normal LSD1 or LSD1K661A.

**Figure 5:**
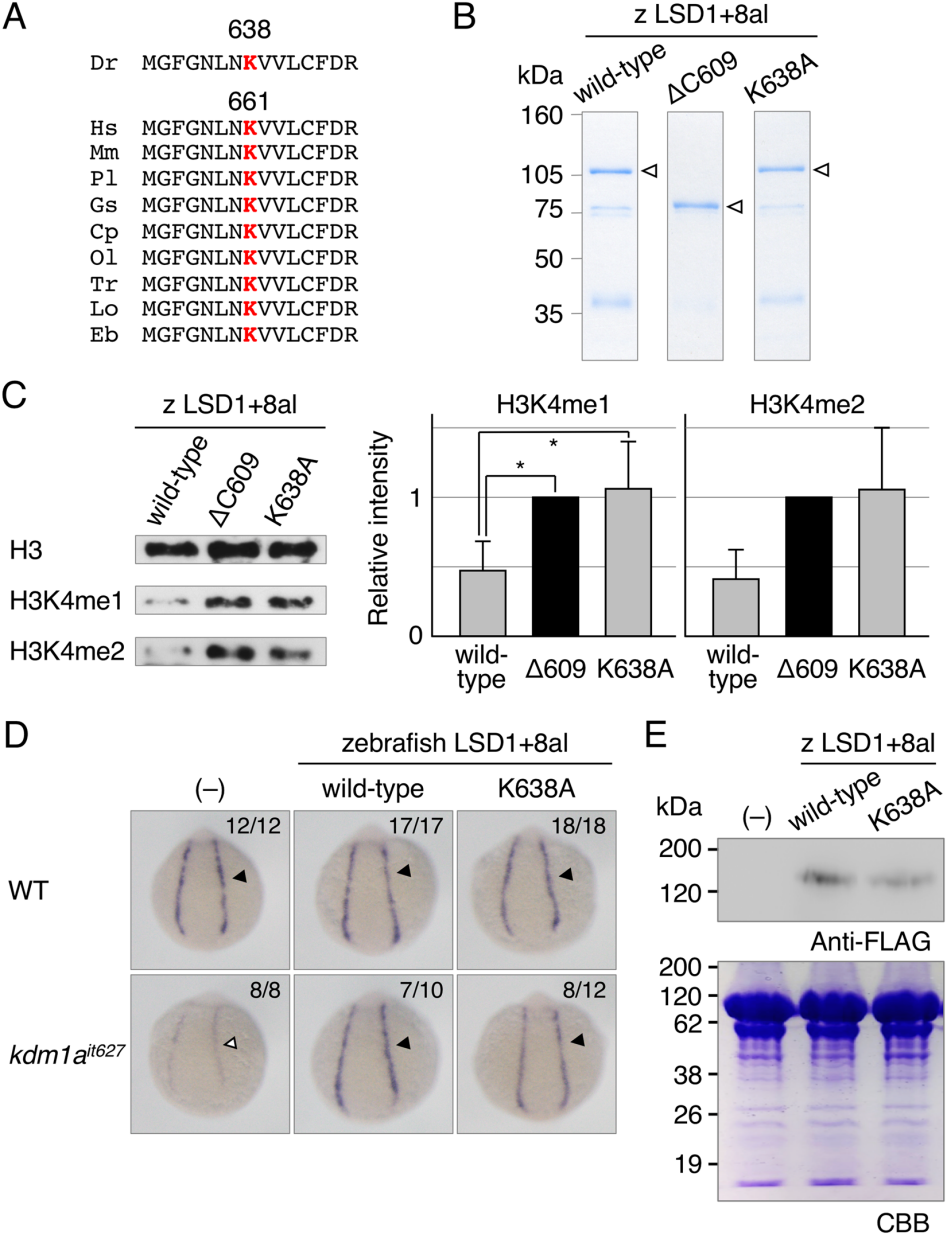
Phenotypic rescue experiments using demethylase-deficient zebrafish LSD1. (**A**) Multiple alignments of vertebrate LSD1 proteins around Lys661 in human LSD1. Red “K” indicates highly conserved lysine residues in the catalytic center of LSD1. (**B**) Bacterially expressed zebrafish LSD1+8al proteins used in a demethylation assay. Arrowheads indicate full-length proteins. (**C**) A demethylation assay of indicated zebrafish LSD1+8al proteins using bulk histones from calf thymus as substrates. Methylated proteins were detected by immunoblotting using specific antibodies. Bar graphs indicate the relative intensity of immunoblot signals standardized to the level of a negative control LSD1+8alΔ609. Error bars represent standard deviations. The experiments was repeated 3 times. Asterisks denote p values < 0.05. (**D**) The *gata1a* expression at 15 hpf in the lateral plate mesoderm (arrowheads) of wild-type or *kdm1a*^*it627*^ embryos injected with or without mRNAs encoding wild-type or K638A mutant-type zebrafish LSD1+8al. (**E**) Immunoblot analysis using anti-FLAG antibody of FLAG-tagged LSD1 proteins in 15-hpf wild-type embryos. Total protein loading was shown by CBB staining.

To verify these observations, we next performed rescue experiments using zebrafish LSD1 constructs. As shown in Fig. 5A, the peptide sequences around Lys661 in human LSD1 are highly conserved among vertebrate LSD1 protein, including in zebrafish. Lys638 is an equivalent amino acid residue in zebrafish LSD1 to Lys661 in human LSD1 protein. We therefore introduced the K638A mutation to LSD1+8al, the most abundant LSD1 variant in zebrafish, and examined its effects in both the *in vitro* demethylation analysis (Fig. 5B,C) and the rescue experiment (Fig. 5D,E). The results showed that the K638A mutation significantly abolished the histone demethylation activity of zebrafish wild-type LSD1+8al, reducing it to a level to that seen in null-mutant LSD1+8alΔC609^13^ (Fig. 5C), but did not alter its rescue effect on the hematopoietic phenotypes in LSD1 mutants (Fig. 5D). An immunoblot analysis showed that the amount of overexpressed LSD1 proteins was equivalent between wild-type and K638A mutant (Fig. 5E). Taken together, these results suggest that the demethylase activity of LSD1 is not required for zebrafish primitive hematopoiesis.

## Discussion

In the present study, we showed that the functions of LSD1 in zebrafish primitive hematopoiesis are splicing variant- and demethylase-independent based on phenotypic rescue experiments, in which the overexpression of any of four human/zebrafish LSD1 splicing variants or enzymatic-deficient human LSD1 K661A/zebrafish LSD1K638A mutant proteins were able to rescue the downregulated *gata1a* expression in LSD1 mutant embryos. Our results also showed that human LSD1 functions in zebrafish embryos in a manner similar to zebrafish LSD1.

K661A is a critical mutation for the demethylation activity of human LSD1 and has been shown to disrupt many physiological functions of LSD1, *e.g*. maintenance of the undifferentiated state of human embryonic stem cells^16^, repression of embryonic stem cell genes^17^, suppression of osteogenic differentiation from human adipose-derived stem cells^18^, activation of telomere nuclease MRE11^19^, promotion of brown fat cell differentiation^20^, stabilization of hypoxia-inducible factor-1α^21^, KU70-dependent suppression of non-homologous recombination repair^22^, inhibition of generating induce pluripotent stem cells^23^, and induction of a stem-like population in sorafenib-resistant hepatocellular carcinoma^24^. However, some reports have described the demethylase-independent functions of LSD1, such as the transcriptional activation of clock genes^25^, stabilization of estrogen-related receptor α in cancer cells^26^, and promotion of proliferation of acute myeloid leukemia in the presence of LSD1 inhibitor^27^. In these cases, the activities of the factors interacting with LSD1 proteins may be important. For instance, Nam *et al*.^25^ showed that making a complex with LSD1 is essential for CLOCK and BMAL1 to transactivate their target genes, while the K661A mutation had no effects on this activity. Similarly, the LSD1 functions in primitive hematopoiesis may require specific-interacting factors that act in a demethylase-independent manner.

We identified splicing variants of LSD1 in zebrafish that have extra peptides at the equivalent sites of mouse and human LSD1+2a and LSD1+8a variants, although the amino acid sequences of the extra peptides differed from those of the mammalian LSD1s. EST databases showed that amino acid sequences of 8a/8a-like peptides are DTVK in mammals/birds/reptiles, FQ in amphibians, and GERCTS/N in fish (see Fig. 1B). Since the peptide sequences around 8a/8a-like are highly homologous between zebrafish and human (exon 8: 100%, 30 amino acids; exon 9: 98%, 48 amino acids), it may be possible to hypothesize that the function of 8a/8a-like is to disrupt these highly conserved regions and their sequences themselves are not important. However, it has been demonstrated that the insertion of DTVK did not change the three-dimensional structure of human LSD1^8^ and that the Thr phosphorylation in DTVK suppressed the enzymatic activity of LSD1+8a^28^. Considering these reports and the highly conservation of the DTVK sequence among mammals/birds/reptiles, amniotes may have acquired new LSD1+8a functions during their evolution. Interestingly, the GERCTS/N sequence in fish is widely conserved not only among teleosts but also in shark (sequence corresponding to GERCTN was found in elephant shark genome (GenBank accession number: KI635890)) and protochordate amphioxus, implying that fish LSD1+8a-like variants may have unique functions different from mammals. Similar discussion can be made for 2a/2a-like, while their peptide sequences vary among fish and do not seem to be important as those in amniotes (see Fig. 1C). Analyses of GERCTS knock-in mice and/or DTVK knock-in zebrafish will provide valuable hints in considering these hypotheses.

Gene expression analyses demonstrated that both zebrafish 2a- and 8a-like peptides are ubiquitously expressed in embryos, larvae, and all tested adult organs. Zebrafish LSD1 variants with 8a-like peptide were expressed in the whole body, unlike human LSD1+8a, which is expressed in a neuron-specific manner^8^. It is possible that biological functions between zebrafish LSD1+8al and mammalian LSD1+8a are different. To clarify this possibility, we would like to generate and analyze zebrafish knockout lines of the 8a-like exon in the future. In contrast to 8a/8a-like variants, the expression profile of zebrafish LSD1 splicing variants containing the 2a-like peptide was similar to that of the human LSD1+2a variant, which is widely expressed in various tissues^8^. LSD1 splicing variants containing the 2a-like peptide were expressed at the highest level in 1-dpf embryos, with its expression tending to decline during embryonic and larval development; this seems to be related to the observations that the expression of LSD1+2a in rat cortex was high in the embryonic brain but drastically decreased as the brain developed^8^, and that the expression in human hematopoietic stem cells (HSCs) was high in quiescent HSCs but decreased in active HSCs and progenitors^11^. LSD1 splicing variants containing the 2a/2a-like peptide may play important roles in embryonic development and/or undifferentiated cells. Generation of zebrafish knockout lines of the 2a-like sequence will also be interesting.

## Methods

### Fish

The AB strain was used as wild-type zebrafish. Genotyping of the LSD1 mutant *kdm1a*^*it627*^ was performed as previously described^13^.

### Plasmids

To construct pCS2FLhLSD1, the full-length human LSD1 cDNA^29^ was subcloned into pCS2FL^30^. cDNAs corresponding to the 2a and 8a peptides of human LSD1 were amplified by PCR using the previously constructed plasmid containing both the 2a and 8a peptides^11^ as templates and inserted into pCS2FLhLSD1 to generate pCS2FLhLSD1+2a, pCS2FhLSD1+8a and pCS2FLhLSD1+2a8a, respectively. To construct pCS2FLlsd1, cDNA sequences corresponding to zebrafish 8a-like peptide was deleted from pCS2FLlsd1+8al^13^ by PCR-based method. For constructing pCS2FLlsd1+2al and pCS2FLlsd1+2al8al, cDNA corresponding the zebrafish 2a-like peptide was amplified by RT-PCR using total RNA from zebrafish embryos at 24 hpf and inserted into pCSFLlsd1 and pCS2FLlsd1+8a, respectively. To construct pCS2FLhLSD1K661A and pCS2FLlsd1+8alK638A, a lysine-to-alanine mutation was introduced by PCR at amino acid 661 in pCS2FLhLSD1 and at amino acid 638 of zebrafish LSD1 in pCS2FLlsd1+8al, respectively. To construct pET15lsd1+8alK638A, cDNA including the mutated site (K638) was amplified by PCR using pCS2FLlsd1+8alK638A as a template, and the amplified fragment was inserted into pET15lsd1+8al (renamed from pET15lsd^13^). pET15lsd1+8alΔC609 was also renamed from previously described pET15lsd1ΔC609^13^.

### Microinjection

Capped mRNAs (500 pg) transcribed from linearized pCS2 plasmids were injected into one-cell-stage embryos as previously described^13^. Overexpressed LSD1 proteins at 15 hpf were detected by immunoblotting using anti-FLAG antibody attached with horseradish peroxidase (A8592, Merck) as previously described^30^. Total protein loading was confirmed by Coomassie brilliant blue (CBB) staining.

### WISH

WISH was performed based on the previous method^13^ with a minor modification in the hybridization temperature (70 °C). Genotyping of the stained embryos was carried out after taking photos with an MZ16 microscope (Leica) equipped with a DP73 digital camera (Olympus).

### RT-PCR

Total RNAs were extracted from the whole body of 1-to-5-dpf embryos and organs from 4-month-old males or 10-month-old females using ISOGEN II (Nippon gene), and cDNAs were synthesized using Rever Tra Ace qPCR RT Master Mix (Toyobo). PCR was performed using KOD FX (Toyobo) and specific pairs of primers: 5′-AGAATGACAGCGAACCTGAG and 5′-GGCGGCCCCTTCTAC (for 2a-like peptide) and 5′-TCTATGAGGCCAATGGACAG and 5′-CTTGCTCTACCATCTCATCC (for 8a-like peptide). Quantitative analysis of RT-PCR was carried out using the ImageJ software program (https://imagej.nih.gov/ij/).

### cDNA cloning

Approximately 800 bp partial cDNAs containing zebrafish LSD1 exon 2- exon 9 were synthesized by RT-PCR using specific primers (5′-GGGGAATTCTCTCGCTAATCTTTCGGAGG and 5′-GGGCTCGAGTGATGTTGCCTCTAACAGGC) and total RNAs extracted from 4 different sources (1-dpf embryos, 5-dpf embryos, adult brain or adult testis) and cloned into the plasmid pBluescript II KS. After transformation into XL1-Blue cells, 48 transformed colonies were randomly picked up and cloned cDNAs were analyzed by PCR using specific primers for 2a-like and 8a-like peptides described above.

### Demethylation

A histone demethylation assay was carried out as described previously^13^. His-tagged LSD1 were synthesized in *Escherichia coli* strain BL21(DE3) using pET plasmids and were analyzed using sodium dodecyl sulfate-polyacrylamide gel electrophoresis followed by CBB staining. Modified histones were assessed by an immunoblot analysis using specific antibodies: anti-monoMeK4H3 (ab8895, Abcam) and anti-diMeK4H3 (UP07–030, Upstate).

### Statistical analysis

A one-way ANOVA was performed followed by Bonferroni’s multiple comparisons test. All statistical analyses were performed using EZR^31^, which is a graphical user interface for R (The R Foundation for Statistical Computing, Vienna, Austria). More precisely, it is a modified version of R commander designed to add statistical functions frequently used in biostatistics.

### Ethics and method statement

All animal experiments were performed in accordance with the animal protocol approved by the Animal Experiment Committee of the University of Tsukuba (Approval Identification Number: 18–042). All methods were carried out in accordance with the Regulation for Animal Experiments in our university and Fundamental Guideline for Proper Conduct of Animal Experiment and Related Activities in Academic Research Institutions under the jurisdiction of the Ministry of Education, Culture, Sports, Science and Technology.

## Supplementary information

Supplementary Information.
