# Supplementary material for: Splicing- and demethylase-independent functions of LSD1 in zebrafish primitive hematopoiesis
Source: Sci Rep. 2020 May 22;10:8521. doi: 10.1038/s41598-020-65428-9 (PMC7244555; doi:10.1038/s41598-020-65428-9)
Supplement: Supplementary file 1 — Supplementary Information. [file 41598_2020_65428_MOESM1_ESM.pdf]

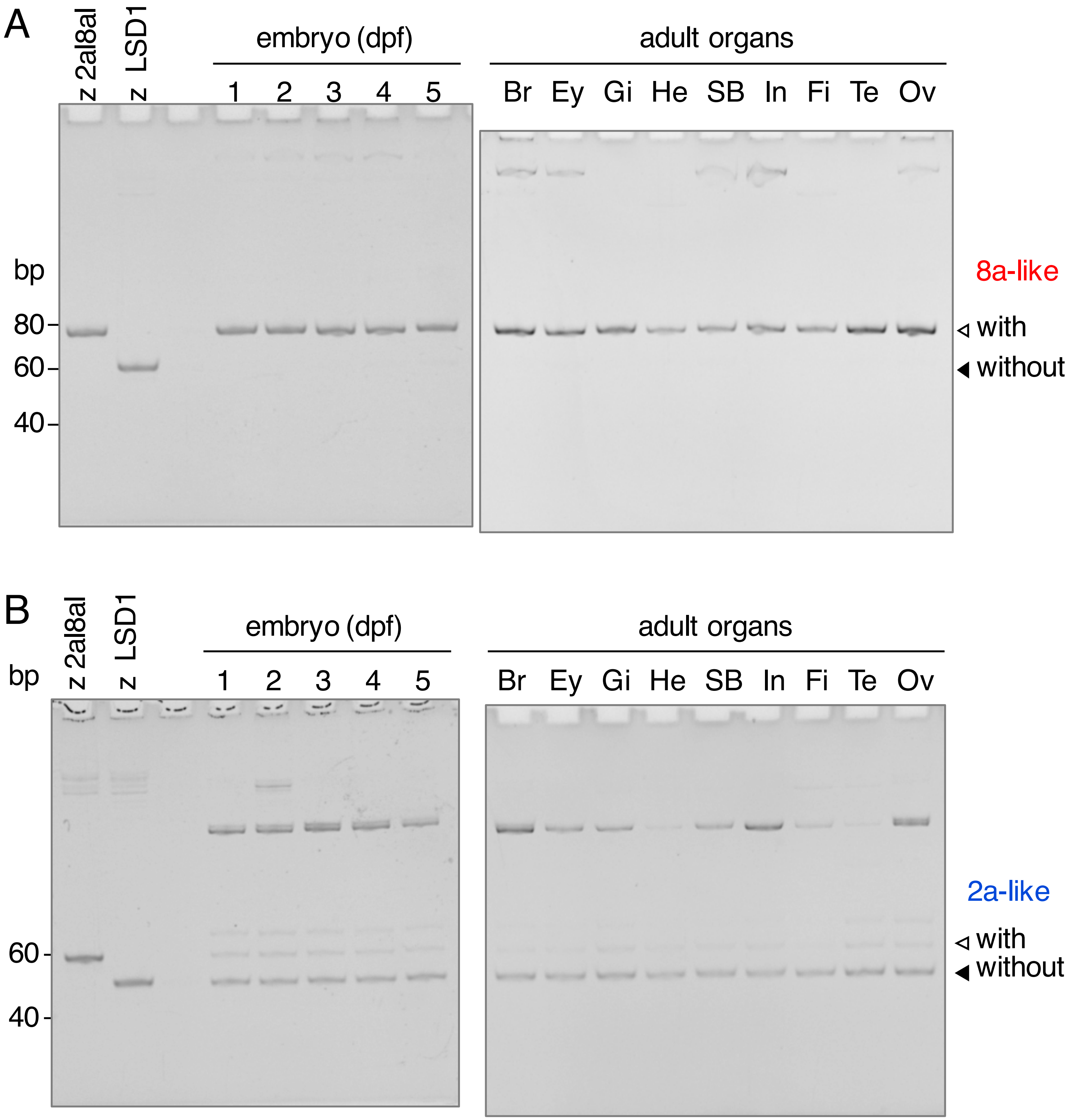

Fig. S2 Tamaoki et al.

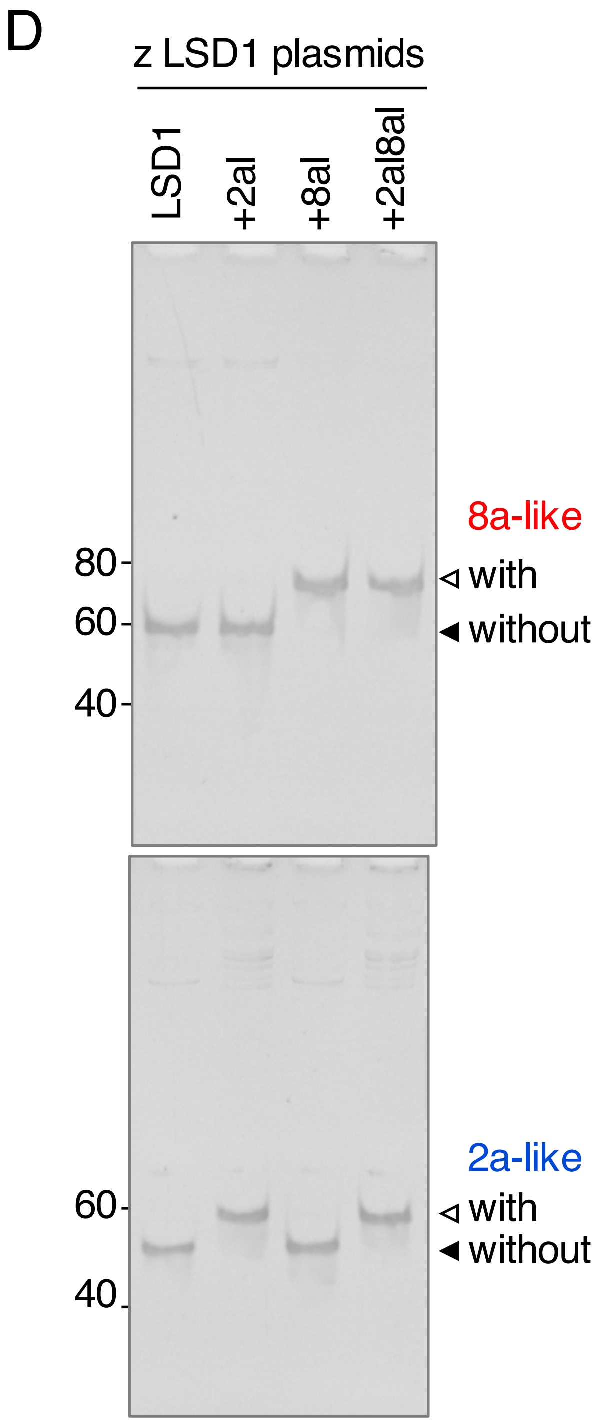

Fig. S2 Tamaoki et al.

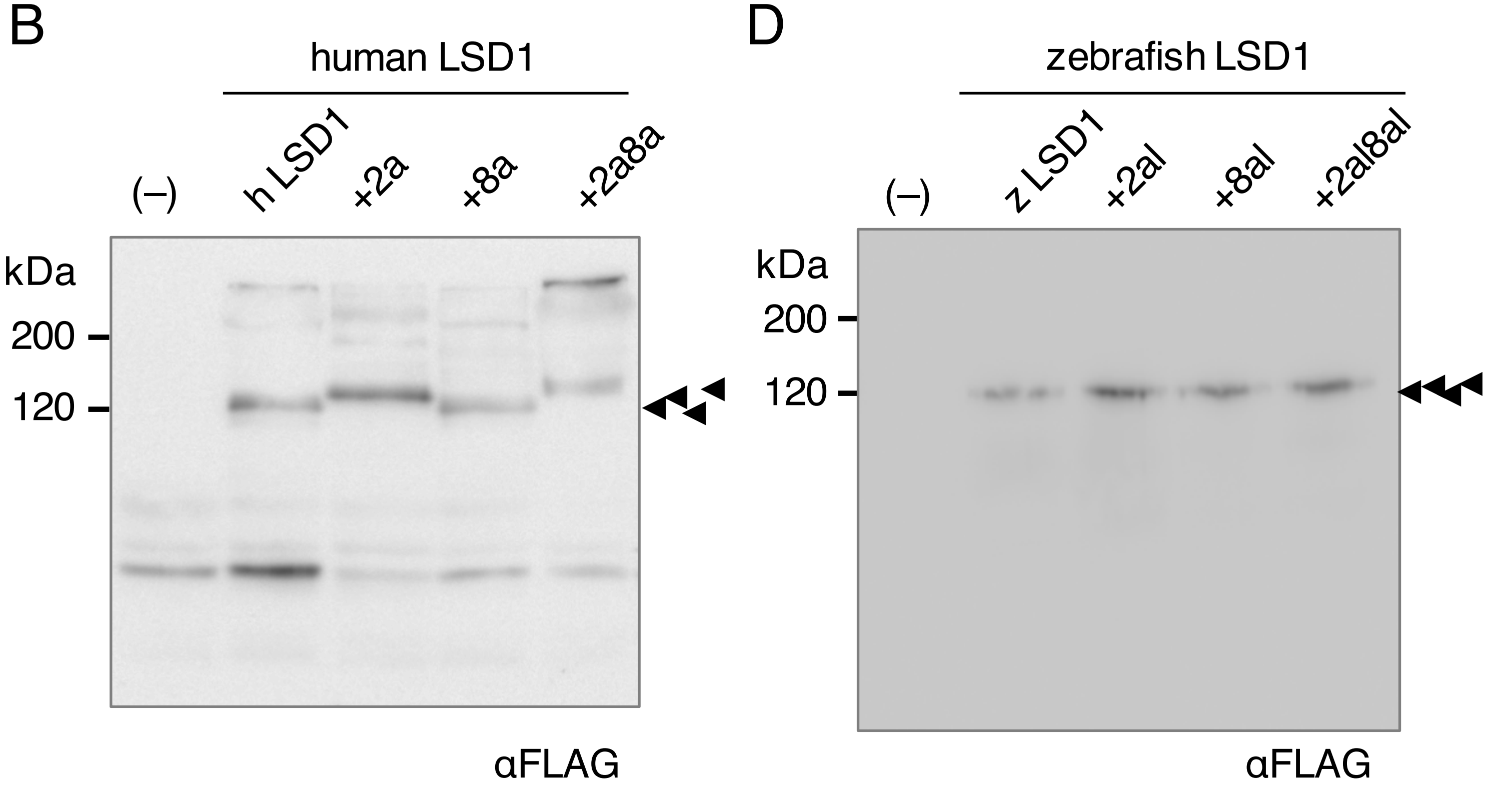

Fig. S3 Tamaoki et al.

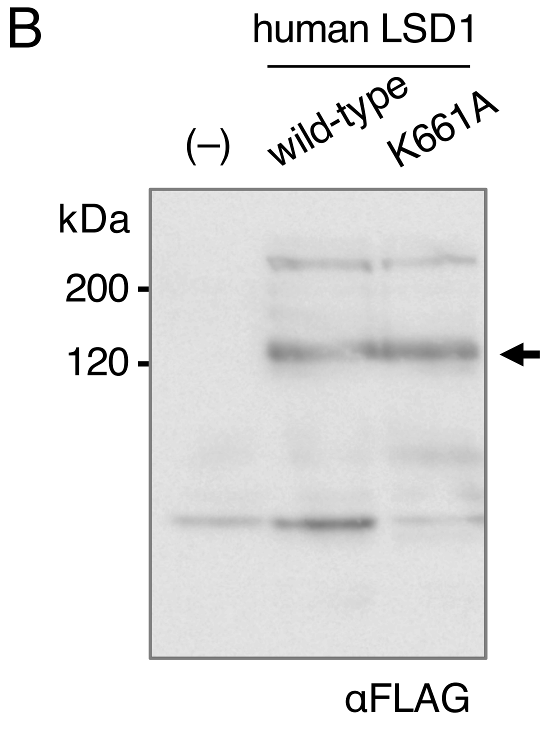

Fig. S4 Tamaoki et al.

C

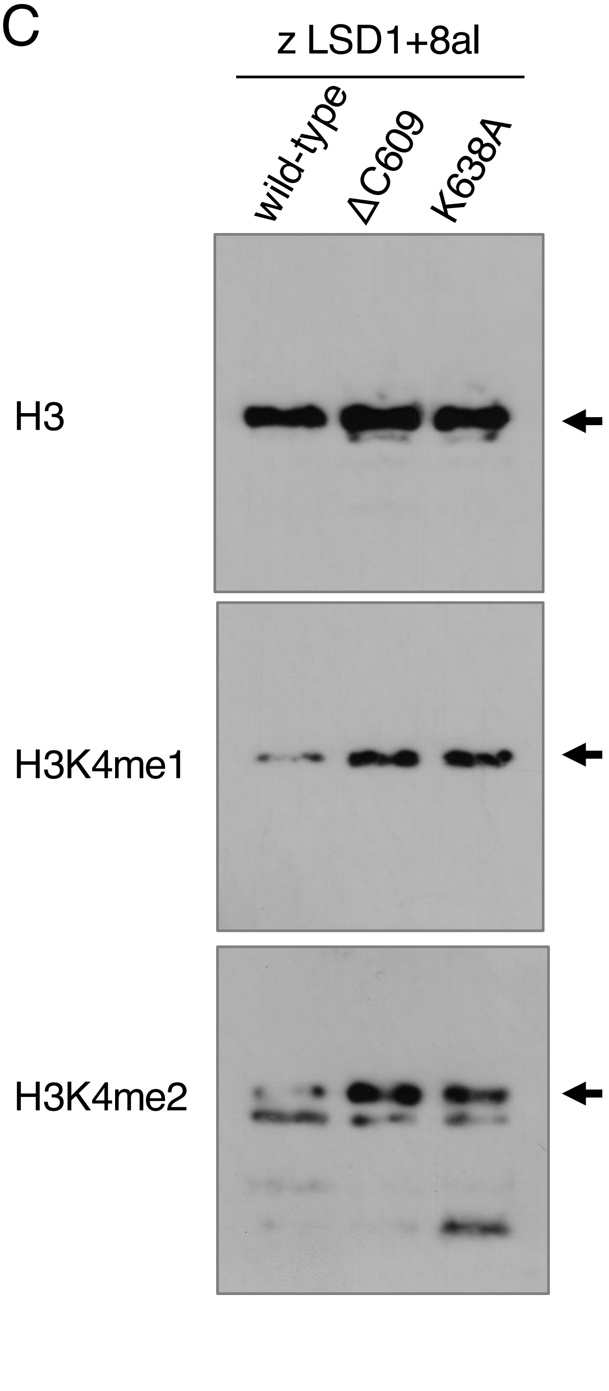

E

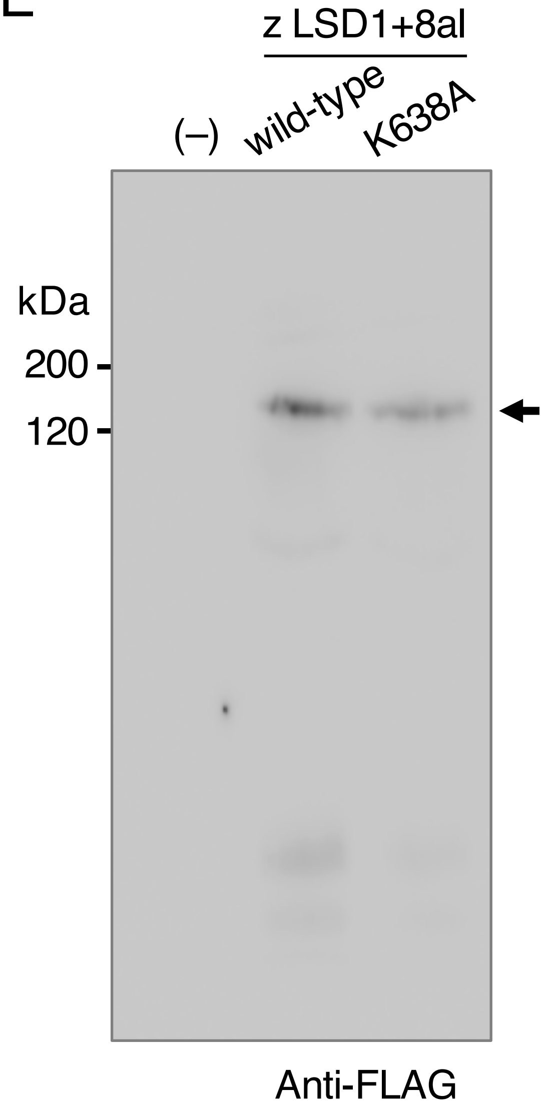

Fig. S5 Tamaoki et al.
